# Matched computed tomography segmentation and demographic data for oropharyngeal cancer radiomics challenges

**DOI:** 10.1038/sdata.2017.77

**Published:** 2017-07-04

**Authors:** MICCAI/M.D. Anderson Cancer Center Head and Neck Quantitative Imaging Working Group, Hesham Elhalawani, Abdallah S.R. Mohamed, Aubrey L. White, James Zafereo, Andrew J. Wong, Joel E. Berends, Shady AboHashem, Bowman Williams, Jeremy M. Aymard, Aasheesh Kanwar, Subha Perni, Crosby D. Rock, Luke Cooksey, Shauna Campbell, Yao Ding, Stephen Y. Lai, Elisabeta G. Marai, David Vock, Guadalupe M. Canahuate, John Freymann, Keyvan Farahani, Jayashree Kalpathy-Cramer, Clifton D. Fuller

**Affiliations:** 1 Department of Radiation Oncology, University of Texas MD Anderson Cancer Center, Houston, Texas 77030, USA; 2 Department of Clinical Oncology, University of Alexandria, Alexandria 21527, Egypt; 3 McGovern Medical School at University of Texas Health Science Center at Houston (UTHealth), Houston, Texas 77030, USA; 4 University of Texas Health Science Center, San Antonio, Texas 78229, USA; 5 Department of Cardiology, Harvard Medical School and Massachusetts General Hospital, Boston, Massachusetts 02115, USA; 6 Furman University, Greenville, South Carolina 29613, USA; 7 Abilene Christian University, Abilene, Texas 79601, USA; 8 Texas Tech University Health Sciences Center School of Medicine, Lubbock, Texas 79905, USA; 9 Columbia College of Physicians and Surgeons, New York, Massachusetts 10032, USA; 10 Texas Tech Health Sciences Center El Paso, Paul L. Foster School of Medicine, Texas 79905, USA; 11 Department of Radiation Oncology, Cleveland Clinic Foundation, Cleveland, Ohio 44124, USA; 12 Department of Imaging Physic, University of Texas MD Anderson Cancer Center, Houston, Texas 77030, USA; 13 Department of Head and Neck Surgery, University of Texas MD Anderson Cancer Center, Houston, Texas 77030, USA; 14 Department of Computer Science, University of Illinois at Chicago, Chicago, Illinois 60607, USA; 15 Department of Biostatistics, University of Minnesota of Public Health, Minneapolis, Minnesota 55455, USA; 16 Department of Electrical & Computer Engineering, University of Iowa, Iowa City, Iowa 52242, USA; 17 Leidos Biomedical Research, Inc, Frederick National Laboratory for Cancer Research, Frederick, Maryland 21701, USA; 18 The Russell H. Morgan Department of Radiology and Radiological Science, Johns Hopkins Medical Institutions, Baltimore, Maryland 21287, USA; 19 National Cancer Institute, National Institutes of Health, Bethesda, Maryland 20892, USA; 20 Department of Radiology and Athinoula A. Martinos Center for Biomedical Imaging, Massachusetts General Hospital and Harvard Medical School, Boston, Massachusetts 02115, USA; 21 Medical Physics Program, University of Texas Graduate School of Biomedical Sciences, Houston, Texas 77030, USA

**Keywords:** Predictive markers, Cancer imaging, Medical imaging, Head and neck cancer

## Abstract

Cancers arising from the oropharynx have become increasingly more studied in the past few years, as they are now epidemic domestically. These tumors are treated with definitive (chemo)radiotherapy, and have local recurrence as a primary mode of clinical failure. Recent data suggest that ‘radiomics’, or extraction of image texture analysis to generate mineable quantitative data from medical images, can reflect phenotypes for various cancers. Several groups have shown that developed radiomic signatures, in head and neck cancers, can be correlated with survival outcomes. This data descriptor defines a repository for head and neck radiomic challenges, executed via a Kaggle in Class platform, in partnership with the MICCAI society 2016 annual meeting.These public challenges were designed to leverage radiomics and/or machine learning workflows to discriminate HPV phenotype in one challenge (HPV status challenge) and to identify patients who will develop a local recurrence in the primary tumor volume in the second one (Local recurrence prediction challenge) in a segmented, clinically curated anonymized oropharyngeal cancer (OPC) data set.

## Background & Summary

Intensity-modulated radiation therapy (IMRT) has evolved in less than two decades to be the state-of-the-art treatment modality for most of the head and neck cancer cases. IMRT is now employed in the treatment of diverse head and neck cancers, in a variety of settings (adjuvant or definitive for the primary disease or re-irradiation for recurrent disease. IMRT is either assigned as a single treatment modality or concurrently with chemotherapy (CRT)^1,2,3^.

The higher therapeutic ratio attained by the application of IMRT in the management of head and neck cancers, especially oropharyngeal cancers, explains the high-esteem to this modality by radiation oncology societies, including the Radiation Therapy Oncology Group (RTOG) which has been endorsing head and neck trials implementing the IMRT modality for years now^4,5^.

With over 20,000 annual cases projected in the U.S, spotlight has been shed on OPC, especially in the era of OPC association to human papilloma virus (HPV)^6^. HPV-associated cancers have been shown to have increased survival and better tumor control with radiotherapy than non-HPV-associated cancers^7^. HPV status is predictive of outcomes, and is tested routinely using immunohistochemistry for p16, a protein, or in situ hybridization for viral DNA^8^. Meanwhile, loco-regional persistence of the disease, recurrence or second primaries following curative intent IMRT-based management remain extremely detrimental^9^. These facts combined have triggered and maintained interest in identifying a subgroup of patients with the lowest risk of disease recurrence after therapy. De-intensification of therapy for this group with subsequent improvement in therapeutic ratio (i.e., similar survival outcomes to those associated with current therapy, along with less toxicity) is among the anticipated payoffs of our study^10^.

**Figure 1:**
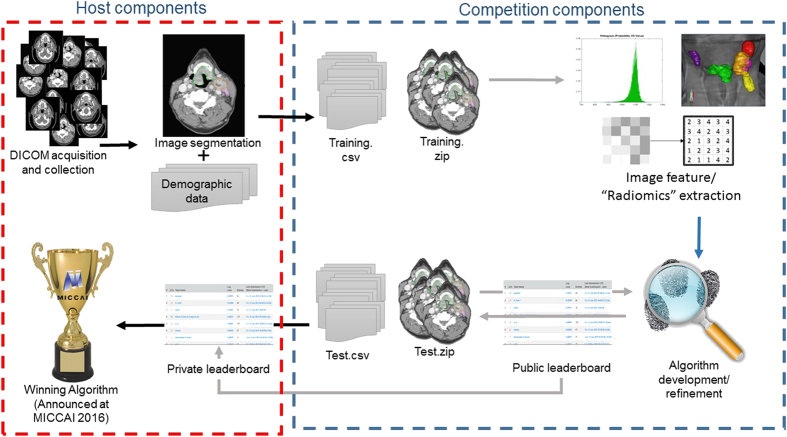
The workflow of the data science competition.

To advance this effort, we prepared these data sets for two machine learning competitions, which were organized by our radiation oncology team at University of Texas MD Anderson Cancer Center (MDACC), as Medical Image Computing and Computer Assisted Intervention (MICCAI) society grand challenges (http://www.miccai.org). Contestants were tasked to predict, using expert-segmented contrast-enhanced computed tomography (CT) images, whether a tumor is HPV positive or negative (as defined by p16 or HPV testing) for the first challenge and the probability of local tumor recurrence for the second challenge. We provided data sets of anonymized Digital Imaging and Communications in Medicine (DICOM) files that represent a relatively uniform cohort of 288 oropharynx cancer patients, supplemented with relevant clinical data, known etiological/biological correlates (specifically, HPV status) as ground truth. Our major target was to assess the ability of participant-developed radiomic workflows to predict binary (phenotypic/genotypic) HPV status and/or possibility of local recurrence, using a defined ‘Training’ cohort as a ‘prior’ data set that includes all input and outcome data, to build up an algorithm. Figure 1 depicts the series of iterative steps for reproducible and consistent extraction of imaging data.

## Methods

### Study population and eligibilty criteria

Diagnostic computed tomography (CT) DICOM files and relevant clinical metadata of 288 patients with histopathologically-proven OPC, treated at our institution between the years 2005 and 2012 were retrospectively restored from a larger oropharynx cohort, using custom-built electronic medical records, ClinicStation (http://www.clinfowiki.org/wiki/index.php/ClinicStation), after an institutional review board (IRB) authorization. Being a HIPAA-compliant retrospective study waived the prerequisite for informed consent. Inclusion entailed the subjects of the study had the following criteria:

1. Histopathologically-proven diagnosis of squamous cell carcinoma (SCC) of the OPC, which encompasses these specified subsites, per the International Classification of Disease, tenth edition (ICD 10): of malignant neoplasm of oropharynx (C10) (http://www.icd10data.com/ICD10CM/Codes/C00-D49/C00-C14/C10-/C10); as detailed in Table 1. We adopted the American ICD-10-CM version.
2. base of tongue (BOT), tonsil, soft palate, pharyngeal wall (posterior and/or lateral), glossopharyngeal sulcus (GPS), vallecula, or other; in case no single subsite of origin could be specified, which is referred to in the ICD 10 coding system as ‘malignant neoplasm of overlapping sites of oropharynx’.
3. Treatment with curative-intent IMRT, which implied that none of these patients had undergone any definitive surgery; prior to the initiation of the radiotherapy treatment course, with subsequent consistent follow-up of >2 years. Also, we didn’t include any alternative radiotherapy techniques other than IMRT, e.g., intensity-modulated proton therapy (IMPT).
4. Known HPV status that was assessed by HPV DNA in situ hybridization^11^ and/or p16 protein expression via immunohistochemistry (IHC).
5. Feasibility of high-quality CT scans, with non-reconstructed axial cuts for each patient, who should have been injected intravenous (IV) contrast material beforehand. Contrast-enhanced axial CT cuts have been the state-of-the-art platform for head and neck target volume delineation for decades; given the higher spatial assimilation of the primary and nodal diseases^12^. However, according to our institutional policy, non-contrast-enhanced CT scans were designated for pre-treatment simulation CT scans. Consequently, we selected from contrast-enhanced CT scans that were primarily ordered for diagnostic purposes.

In accordance with these inclusion criteria, OPC patients who were non-SCC (3 individuals) or had unknown HPV status (591 individuals) were excluded. Furthermore, even qualified patients, whose CT scan features didn’t harmonize with our eligibility criteria, were excluded. Toward this end, 3 patients were excluded due to a lack of pre-IMRT CT scans, and 11 patients were excluded because their pre-IMRT CT scans were non-contrast-enhanced. An additional 11 patients were also excluded due to inadequacy of the attained CT cuts, specifically, artifacts masking the region of interest stemming from metal dental fillings or CT cuts that didn’t encompass the entire ROI. Moreover, it was found that poor CT timing was a cause for exclusion, i.e., an inability to accurately depict the real magnitude of the primary and nodal disease at time of diagnosis. Fifteen individuals had received induction chemotherapy with no available earlier CT scans, while an additional 21 individuals had undergone excisional biopsies of the primary disease (e.g., tonsillectomy) or suspicious lymph nodes with no CT scans performed in advance. This resulted in a net result of 315 OPC patients, who constituted our ultimate competition cohort.

**Figure 2:**
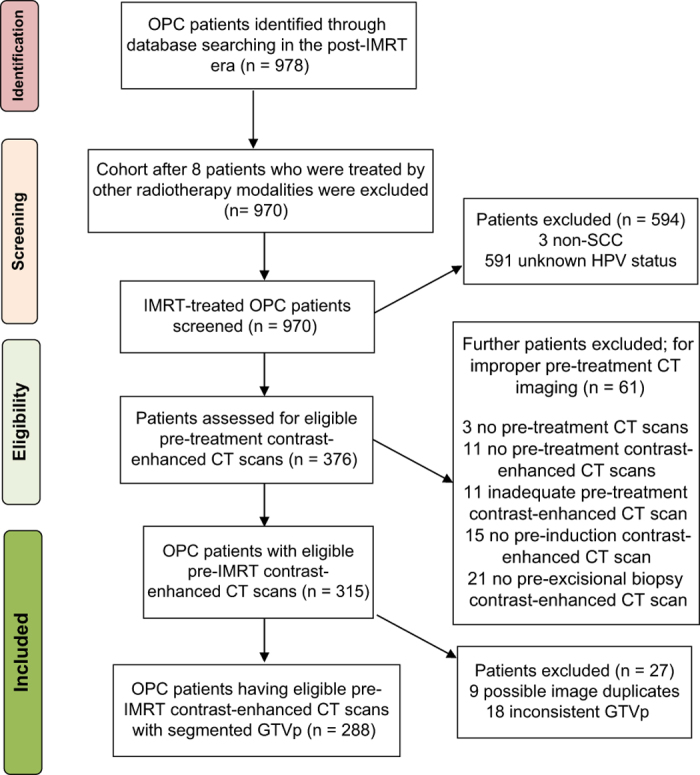
Flowchart of patient selection for inclusion.

However, as a part of our team’s systematic process of checking the competition, it was discovered that there had been 9 incidences of duplicate images while rendering corresponding DICOM-RT files, which could not be amended. Moreover, the segmented GTVp in 18 patients wasn’t adequately representative of the primary tumor gross volume. Accordingly, after omitting these 27 patients’ files, the data set described in this article encompasses 288 patients, as mapped in Fig. 2.

We imported the contrast-enhanced CT scans from the patients’ electronic medical records that were performed not only before the initiation of the radiation treatment course, but also preceding any significant alteration in the disease, e.g., induction chemotherapy or excisional biopsies. The yielded CT scans of choice were imported to VelocityAI 3.0.1 software (powered by VelocityGrid; http://www.velocitymedical.com/), our institutionally-adopted contouring platform, which was used by two expert radiation oncologists to segment our ROIs, namely the pre-treatment gross tumor volume (GTV), both of the primary disease (GTVp) and the metastatic lymph nodes (GTVn).

Both the segmented structures, along with the relevant clinical meta-data extracted from the patients’ profiles were the pillars for our radiomics challenges. Defined as deriving quantitative imaging features from routine imaging data through a multi-step image processing, radiomic analyses have been implemented in correlation with clinical data to generate promising meaningful data; that can be further projected into prognostic and/or predictive non-invasive biomarkers^13,14,15^.

Hence, our team constructed two public challenges examining radiomic analytics for head and neck cancer applications, specifically for the OPC domain. These public challenges were a part of a spate of activities related to the computational precision medicine satellite activities, supported by MICCAI society. They were designed to allow any and all data science teams to test their radiomic analysis skills, in order to discriminate etiologic features and treatment outcomes of patients in a clinically curated anonymized OPC data set (*n*=288) with contrast-enhanced CT-scans and standardized radiation oncologist-segmented primary tumor and nodal volumes.

Challenge 1 evaluated competitor's ability to classify HPV/p16 status (HPV status challenge) (http://inclass.kaggle.com/c/oropharynx-radiomics-hpv), while Challenge 2 sought to predict which patients will have a local recurrence in the primary tumor volume (Local recurrence prediction challenge) (https://inclass.kaggle.com/c/opc-recurrence). Both challenges were hosted online at the machine learning challenge website Kaggle in Class (https://inclass.kaggle.com).

### Patient demographics and clinical end points

In this data set, the records of the included 288 patients with OPC treated with curative-intent IMRT at The University of Texas MD Anderson Cancer Center, drawn from a larger oropharynx cohort between the years 2005 and 2012 were thoroughly screened for specific demographic data, disease characteristics, treatment details and outcomes^16,17^. Table 2 includes Supplementary Information about the data provided for this cohort of patients. Collective clinical characteristics of the patients, disease and treatment are given in Supplementary Table 1.

The patients’ demographics data were provided the same format for both challenges. These included: gender, age at diagnosis and race. Disease characteristics encompassed: tumor laterality and oropharynx subsite of origin. Furthermore, TNM (Tumor, node and metastases) classification was also provided, where T category describes the original (primary) tumor, as regard its size and extent, per the American Joint Committee on Caner (AJCC) and Union for International Cancer Control (UICC) cancer staging system, 7th edition^18^ (https://cancerstaging.org/references-tools/Pages/What-is-Cancer-Staging.aspx). Noteworthy, patients with Tx, i.e., primary tumor couldn’t be assessed, were normally excluded. Similarly, the N category describes whether or not the cancer has reached nearby lymph nodes, per the AJCC and UICC cancer staging system, 7th edition, along with the corresponding AJCC stage. Tumor histology and grade of differentiation were evaluated by pathologists at the parent institution, whereas for patients diagnosed at an outside healthcare facility, central pathology review was performed. Smoking status at diagnosis was recorded, per the 2016/2017 ICD 10 definitions as categorized in Table 3.

This was followed by individual smoking-pack years, which represents an equivalent numerical value of lifetime tobacco exposure. A pack year is defined as twenty cigarettes smoked every day for one year. We used an online calculator (http://smokingpackyears.com/) whenever unfeasible to calculate tobacco exposure (e.g., when oral tobacco ‘dips’ were not quantified); we used the coding ‘NA’ (i.e., ‘not assessable’).

For the ‘Local recurrence prediction challenge’, additional details were provided as well. These included:

- Radiation treatment course duration, which was precisely reported in days given the well-known fact of increased incidence of local failures as a function of a protracted radiation time, while managing head and neck cancers^19^.
- Total dose of irradiation each patient received in Grays^20^.
- Total number of daily radiation treatment fractions. (Tabular summary of the radiotherapy data is presented in Table 4).
- The addition of systemic treatment (whether cytotoxic or targeted; single or in combination) was reported dichotomously (yes or no), both during the induction phase (i.e., before the initiation of radiation treatment course) and the radiation treatment (i.e., during the course of radiation treatment, simultaneously). Also, individual patient’s vital status was dichotomously reported as ‘1=alive’ or ‘0=dead’; as an indicator for overall survival status. Finally, for the ‘HPV status challenge’, HPV status was offered in the training data set as ‘positive’ or ‘negative’ and left unknown for the test data set, and similarly the occurrence of ‘local tumor recurrence’ was provided for the training set only in the ‘Local recurrence prediction challenge’ as ‘1=primary tumor recurrence’ or ‘0=no primary tumor recurrence’, while kept unknown for the test data set for the sake of the challenge. Local recurrence was defined as evidence of recurrent neoplastic disease within the same subsite or other subsites of the oropharynx^21^.

### Treatment strategy and IMRT technique:

Multidisciplinary schematic treatment approach was meticulously detailed by Garden *et al.*^9,22^ along with MD Anderson Cancer Center protocols of trials studying the implementation of IMRT in locally advanced oropharyngeal cancer, e.g., NCT01893307 (https://clinicaltrials.gov/ct2/show/NCT01893307?term=NCT01893307&rank=1). Assessment of an oropharyngeal tumor starts with a global history and physical examination. Typically, this is followed by nasopharyngolaryngoscopy procedure with biopsies of suspicious zones. The vast majority of patients had contrast-enhanced CT scans of the head and neck performed for the purpose of diagnosis and staging of oropharyngeal cancer, whereas some of them underwent other imaging modalities, like magnetic resonance imaging (MRI) and/or positron emission tomography-computed tomography (PET-CT). An institutional transdisciplinary team adopted the comprehensive management approach for all patients at a tumor board, held on weekly basis. Surgery was mostly implemented for diagnostic purposes, preceding radiotherapy. Neck dissection after radiation was reserved for cases, where complete clinical response couldn’t be achieved, mainly estimated by physical examination, computed tomography, and ultrasonography. The selection of the eligible patients for systemic treatment and the prescribed regimens was determined according to the extent of the disease, performance status and associated comorbidities. Consequently, patients with heavy primary tumor disease burden and/or sizable lymph nodes were routinely assigned concurrent chemoradiation. Given the well-established correlation between advanced nodal disease and increased incidence of distant recurrence^23^, this group of patients were usually prescribed an upfront induction chemotherapy.

Pinnacle planning system (v4 through v9, Philips Medical Systems, Andover, MA) was employed in radiotherapy treatment planning. Treatment was delivered with a static gantry approach. Patients treated to only 1 side of the neck were typically planned with a template using 7 beams equidistant through a 190° arc, whereas the template for patients treated to both sides of the neck used 9 beams set equidistant through 360°. Beam angles and number were reshaped during the optimization process. In general, IMRT was used to treat the primary tumor and upper neck nodes. The isocenter was mostly set above the arytenoids, and IMRT was delivered to portals above the isocenter, whereas the lower neck below the isocenter was treated with an anterior beam, with a larynx and/or full midline block in most cases. Nodes in levels 3 to 4 were boosted with glancing photon beams and/or electron beams. Additionally, bulky nodes in the IMRT fields were occasionally boosted with electrons. A ‘whole-field’ IMRT approach was regularly used in situations in which the patient's anatomy or primary tumor location created concerns that tumor might be underdosed using the ‘split-field’ approach^24^. IMRT was delivered using Varian (Varian Medical Systems, Palo Alto CA) linear accelerators delivering 6-MV photons.

Target volumes were individualized. After simulation, contours of the target volumes were delineated and reviewed in our quality assurance clinic as described elsewhere in details. Rosenthal *et al.* established the necessity for a comprehensive peer review planning clinic, being an integral component of IMRT quality assurance^25^. Gross disease with an 8–10 mm margin was defined as CTV1. Treatment was prescribed to CTV1. A planning target volume (PTV) of 3 mm was generated around each clinical target volume (CTV). One or 2 CTVs were created to encompass subclinical disease, including additional margin on CTV1, anatomic sites of potential direct spread, and uninvolved levels of the neck at risk. The spinal cord was limited to maximum 45 Gy. The brainstem was typically limited to 50 Gy, but taking into consideration beam path toxicities, stricter constraints were placed^26^. The goal set for the parotid glands was regularly a mean dose of ≤ 26 Gy, though the clinical setting and proximity of the parotid to gross nodal disease influenced the priority placed on this goal.

### HPV detection

All tumors were tested for their HPV status via: evaluating the presence of HPV16 DNA by use of the in situ hybridization-catalyzed signal amplification method for biotinylated probes and/or the expression status of p16 via immunohistochemistry (IHC)^27^. Recent meta-analysis has shown that the proportion of HPV-associated OPC has jumped dramatically worldwide from 40.5% in studies enrolling patients before 2000 to 72.2% in studies recruiting patients after 2005 and our cohort showed similar trend^28^. In case any discordance between HPV DNA and p16 testing results was encountered, the p16 status was utilized to attribute HPV status, attributed to the fact that p16 positivity can encompass a larger number of HPV strains than in situ hybridization^29^. Table 5 details HPV status testing.

### Imaging characteristics

Contrast-enhanced CT images were restored from the patients’ electronic medical record, with a section thickness of 1–5 mm (median: 1.25 mm in 84.7% of the cases) and an X-ray tube current of 100–584 mA (220 mA for 68.1% of the patients) at 100–154 kVp (120 kVp for 66% of the patients). Most of the CT scans (92%) were obtained using GE Medical Systems scanners, specifically LightSpeed16 (55.2%) and LightSpeed VCT (27.4%) models.

Display field of view was 25 cm; axial images were acquired by using a matrix of 512×512 pixels and reconstructed with a voxel size of 0.048828 cm×0. 048828 cm along the x- and y-axis. Forty-four patients had CT scans with a slice thickness (Z-dimension) that was not equal to 1.25 mm (range 0.5 to 5 mm). One hundred twenty milliliters of contrast material were injected at a rate of 3 ml sec^−1^, followed by scanning after a 90-second delay. Detailed acquisition parameters are provided in Supplementary Table 2, including full imaging specifications for each DICOM file, scanner manufacturer and software details, along with CT protocol.

### Manual segmentation of regions of interest (ROIs)

Gross tumor volumes (GTV) for primary tumor (GTVp) constituted our regions of interest for this project. Gross nodal tumor volumes (GTVn) were additionally segmented, to help give the contestants a better idea about the extent of disease. Gross tumor volumes were defined as per ICRU 62/83, specifically, ‘the gross demonstrable extent and location of the tumor’^30^. In case of multiple separate metastatic lymph nodes, gross nodal tumor volume (GTVn) were numbered separately, starting from the most superior node, which was given the name (GTVn1), then (GTVn2), *etc*. No GTVn was segmented in case of node negative (N0) disease or CT scan was performed following a lymph node excisional biopsy.

Tumor volumes were manually segmented on each individual patient’s diagnostic contrast-enhanced CT axial images and simulation CT images by the collaborators independently. They were blinded to relevant clinical meta-data and their segmentation was revised by a radiation oncologist (HE), along the regulations we followed for previous projects^21^. The segmentation process was governed by the guidelines of the International Commission on Radiation Units and Measurements (ICRU) report 83. Segmentation primarily relied on the findings from physical examination, fiberoptic nasopharyngolaryngoscopy and imaging studies. Manual segmentation was performed by using commercial treatment planning software VelocityAI 3.0.1 software.

### Data de-identification

**Figure 3:**
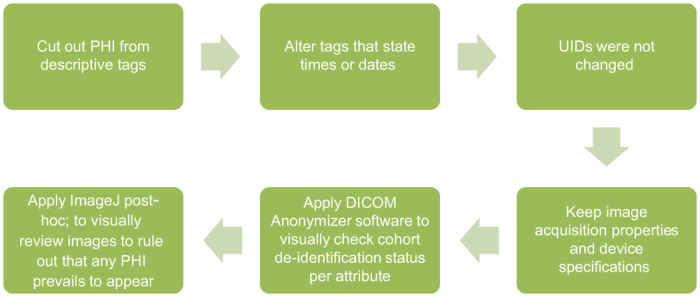
Work flow of DICOM PHI anonymization.

We used an open-source tool to de-identify DICOM files, DICOM Anonymizer version 1.1.6.1 (https://sourceforge.net/projects/dicomanonymizer/). This program is designed to replace the patient tags in all the DICOM files in a folder (and sub-folders) with other strings assigned. It neither changes the length of the DICOM tag nor alters Unique Identifiers (UIDs). The following DICOM tags were de-identified: patient’s name, patient’s identifier (ID), patient’s birth date, study description, manufacturer, instance creation date, instance creation time, study date, series date, acquisition date, image date, performed procedure step start date, accession number and study ID^31^. These DICOM tags were chosen based on a custom confidentiality profile that we’ve adopted in accordance with the Health Insurance Portability and Accountability Act (HIPAA), as designated by the DICOM standards committee Attribute Confidentiality Profile (DICOM PS 3.15: Appendix E; ftp://medical.nema.org/medical/dicom/2011/11_15pu.pdf), which describes a standard procedure and documentation for removal of protected health information (PHI) from DICOM images^32^. Table 6 depicts the PHI tags, embedded in the DICOM metadata tags that were de-identified. A final DICOM de-identification quality assurance was applied using a software, named ImageJ (https://imagej.nih.gov/ij/), which collects attributes per patient in a report that was scanned to guarantee optimal anonymization accomplishment by the implemented DICOM anonymizer software. Figure 3 portrays DICOM de-identification workflow.

### Competition leaderboard

**Figure 4:**
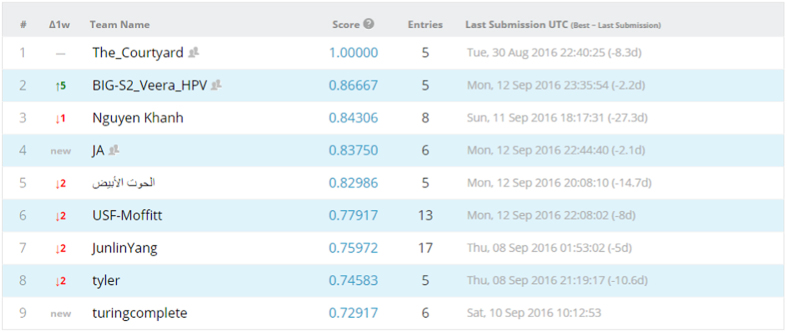
Final public leaderboard for the HPV status challenge.

**Figure 5:**
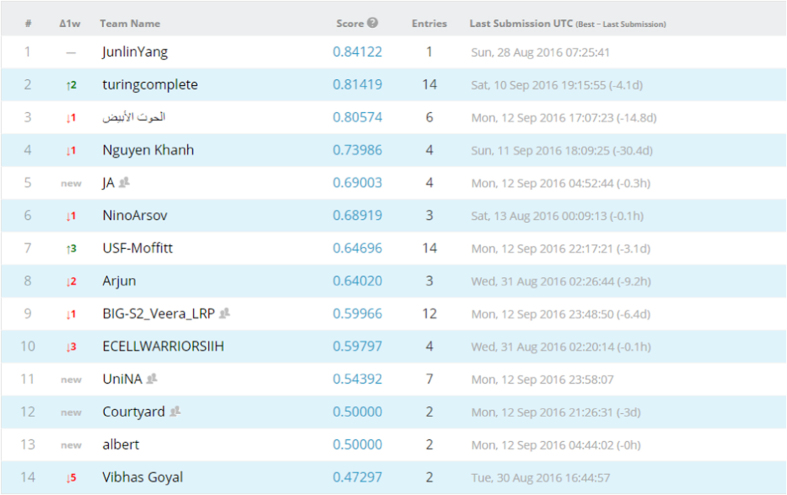
Final public leaderboard for the Local recurrence prediction challenge.

**Figure 6:**
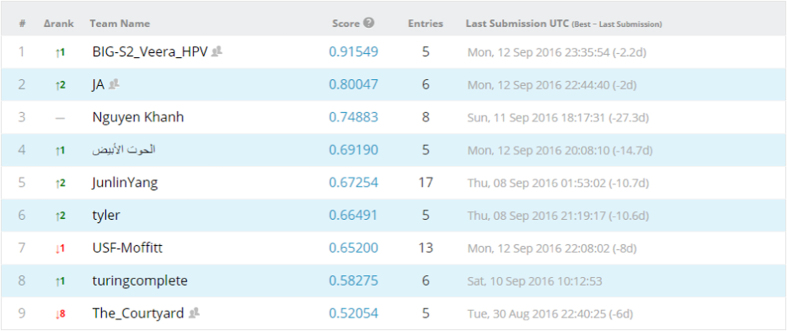
Private leaderboard for the ‘HPV status challenge’.

**Figure 7:**
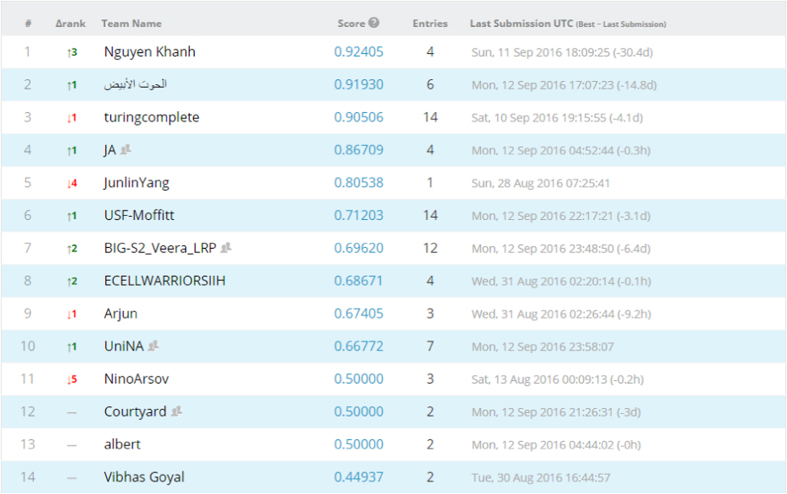
Private leaderboard for the ‘Local recurrence prediction challenge’.

We opted to run both competitions as public competitions, where anyone can participate. We then set the evaluation metric of both competitions to be area under receiver operating characteristic curve (AUC). We divided our data set into training and test data sets, evenly split according to outcome classes, i.e., HPV status/local recurrences, with separate CSV files and DICOM folders. For the test set, the outcome column was obscured. Patients, disease and treatment characteristics for the training and test sets are tabulated in Supplementary Tables, Supplementary Tables 3 and 4. Afterwards, Kaggle in Class further split the test set into private and public subsets, each scored separately. Results for the public records appear in the ‘public leaderboard’, which shows some relative performance during the competitions that was continuously updated calculated on contestants’ submissions. Figures 4 and 5 depict how the final ‘public leaderboard’ for the ‘HPV status challenge’ and the ‘Local recurrence prediction challenge’ looked, respectively. Kaggle in Class administration withholds the answers for this data set to compare against the competitors’ predictions. When the competition was over, each competitor’s top submission was selected and evaluated based on the remaining portion of the test set that was kept hidden from the competitors till the end, or the private fraction. Competitors were never sent a feedback about their scores on this portion, so it is the ‘private leaderboard’. Final competition results were derived from the ‘private leaderboard’, and the winner was the person or team at the top of the ‘private leaderboard’; to eliminate the possibility a model that overfits to the specific noise in that data. Figures 6 and 7 illustrate the ‘private leaderboard for ‘HPV status challenge’ and ‘Local recurrence prediction challenge’, respectively.

### Competitions

Contestants were invited to download the DICOM-RT files, along with clinical meta-data tables, with subsequent mechanistic analysis, that includes the performance of individual risk assessments. The region of interest for robust texture analysis was the primary gross tumor volume, which is denoted as GTVp. We also provided segmented gross nodal tumor volume or the GTVn, for future projects that will dig into potential predictive radiomic biomarkers, indicative of patterns of failure. The ultimate goal was the development of an algorithm that yields the HPV status or the probability of local failure in OPC patients, based on their particular radiomic signatures. All the previous steps constituted the workflow of our dedicated machine learning projects, as previously depicted in Fig. 1.

The winning algorithms were presented by the winners at the full-day CPM satellite workshop, as a part of the program of MICCAI 2016 (http://www.miccai2016.org/en/). The top-winners from each challenge were invited to share their approach and algorithm to the ‘Data Science’ community *via* an online video conferencing.

### Code availability

- **DICOM Anonymizer version 1.1.6.1** is the open-source tool we used to de-identify DICOM files. The code for this tool is available online and readily accessible at https://sourceforge.net/p/dicomanonymizer/code/HEAD/tree/
- **ImageJ**, a free software offered by the National Institutes of Health, USA, as a public domain Java processing program. The code for the software is accessible at: https://imagej.nih.gov/ij/.
- Smoking pack-years were computed using an **online calculator** helps to produce a numerical value of lifetime tobacco exposure, openly accessible at http://smokingpackyears.com/.

## Data Records

This data descriptor describes data that were used for head and neck radiomics challenges, designed for teams involved in machine learning to test their ability to leverage radiomics and/or machine learning workflows. This OPC data set (*n*=288) encompasses anonymized clinically curated contrast-enhanced CT scans (73,230 DICOM-RT files, including 288 DICOM-STRUCT files) that show primary tumor and nodal disease as segmented by expert radiation oncologists. The 2 challenges were a part of a spate of activities related to the Computational Precision Medicine (CPM) satellite activities at MICCAI 2016 (http://www.cpm-miccai.org) hosted at the machine learning challenge website Kaggle in Class. Data is also available from figshare (Data Citation 1 and Data Citation 2).

Relevant clinical meta-data files are also provided as.csv sheets. Table 7 details the various data records, along with a brief description. The CT images follow the standard DICOM format are organized by anonymized patient ID number (Patient_ID), and can be cross-referenced against the data table using this identifier.

## Technical Validation

- **Pinnacle treatment planning system** (Philips Radiation Oncology Systems, Fitchburg, WI) engages a collapsed cone convolution (CCC) algorithm, for optimal dose calculation^33^.
- **ClinicStation (Electronic Medical Record System)**, a custom-built electronic medical record system by MDACC, that started in 1999 with subsequent significant improvement in 2007 that allowed further new capabilities, as integrating research data and accessing data from virtually every electronic source within the institution, among others, thus serving great role in patient care and research. http://www.clinfowiki.org/wiki/index.php/ClinicStation
- **VelocityAI 3.0.1 software** (powered by VelocityGrid), our institutionally-adopted contouring platform, was used for segmenting ROIs. http://www.velocitymedical.com/

## Usage Notes

DICOM, as a standard platform for managing medical images and related information, is indispensable to radiation oncology workflow^34^. As a consequence, various radiotherapy-specific DICOM objects (i.e., DICOM-RT) were created, e.g., DICOM-STRUCT which refers to DICOM structure set, among others. Various validated open-source softwares that can be applied as texture analysis toolboxes^35^.

Here are some of the most commonly used computational resources:

- **IBEX** (Imaging Biomarker Explorer)—an open-source platform for image feature extraction, primarily developed using MATLAB and C/C++ programing languages; http://www.ibex-lib.org/download
- **MazDa**—a computer program for calculation of texture parameters, written in C++ and Delphi©; http://www.eletel.p.lodz.pl/programy/mazda/index.php?action=mazda
- **CGITA**—another open-source texture analysis toolbox, built in the MATLAB environment; http://code.google.com/p/cgita

## Additional Information

**How to cite this article:** Elhalawani, H. *et al.* Matched computed tomography segmentation and demographic data for oropharyngeal cancer radiomics challenges. *Sci. Data* 4:170077 doi: 10.1038/sdata.2017.77 (2017).

**Publisher’s note:** Springer Nature remains neutral with regard to jurisdictional claims in published maps and institutional affiliations.

## Supplementary Material



Supplementary Information

## Figures and Tables

**Table 1:** Oropharynx cancer subsites of origin included in this data set.

**ICD 10 nomenclature of oropharynx subsites of origin**	**Applicable to**	**ICD 10 code**	**Hyperlink to the ICD 10 website**
Malignant neoplasm of tonsil	Malignant neoplasms of tonsillar fossa, tonsillar pillars (anterior and/or posterior), overlapping sites of tonsil or tonsil (unspecified)	C09	http://www.icd10data.com/ICD10CM/Codes/C00-D49/C00-C14/C09-/C09
Malignant neoplasm of base of tongue	Malignant neoplasms of dorsal surface of base of tongue/ fixed part of tongue NOS/ posterior third of tongue.	C01	http://www.icd10data.com/ICD10CM/Codes/C00-D49/C00-C14/C01-
Malignant neoplasm of soft palate		C05.1	http://www.icd10data.com/ICD10CM/Codes/C00-D49/C00-C14/C05-/C05.1
Malignant neoplasm of glossopharyngeal sulcus		C09.0	http://icd10coded.com/cm/neoplasm/?page=14
Malignant neoplasm of vallecula		C10.0	http://www.icd10data.com/ICD10CM/Codes/C00-D49/C00-C14/C10-/C10.0
Malignant neoplasm of lateral wall of oropharynx		C10.2	http://www.icd10data.com/ICD10CM/Codes/C00-D49/C00-C14/C10-/C10.2
Malignant neoplasm of posterior wall of oropharynx		C10.3	http://www.icd10data.com/ICD10CM/Codes/C00-D49/C00-C14/C10-/C10.3
Malignant neoplasm of overlapping sites of oropharynx		C10.8	http://www.icd10data.com/ICD10CM/Codes/C00-D49/C00-C14/C10-/C10.8

**Table 2:** Supplementary Information about the data provided for both challenges.

**Data category**	**Description**
Patient ID	Numbers_given_randomly_to_the_patient_after_anonymizing_the_DICOM_PHI_tag_(0010,0020;_Patient_ID)
HPV/p16 status	Human Papilloma Virus status, as assessed by HPV DNA in situ hybridization^11^ and/or p16 protein expression via immunohistochemistry (IHC), with the results described as: 1 (i.e., Positive) or 0 (i.e., Negative)
Gender	Patient's sex
Age at diagnosis	Patient's age in years at the time of diagnosis
Race	American Indian/Alaska Native, Asian, Black, Hispanic, White or NA (Not applicable)
Tumor laterality	Right, left, bilateral
Oropharynx subsite of origin	The subsite of the tumor within the oropharynx, i.e., base of tongue (BOT), tonsil/soft palate/pharyngeal wall/glossopharyngeal sulcus (GPS)/other (no single subsite of origin could be identified)
T category	The T category describes the original (primary) tumor, as regard its size and extent, per the American Joint Committee on Caner (AJCC) and Union for International Cancer Control (UICC) cancer staging system. It could be T1, T2, T3, T4. https://cancerstaging.org/references-tools/Pages/What-is-Cancer-Staging.aspx
N category	The N category describes whether or not the cancer has reached nearby lymph nodes, per the AJCC and UICC cancer staging system. It can be N0, N1, N2a, N2b, N2c or N3. https://cancerstaging.org/references-tools/Pages/What-is-Cancer-Staging.aspx
AJCC Stage	AJCC cancer stage. https://cancerstaging.org/references-tools/Pages/What-is-Cancer-Staging.aspx
Pathological grade	The grade of tumor differentiation. It is described as: I, II, III, IV, I-II, II-III or NA (Not assessable)
Smoking status at diagnosis	Never, current, or former
Smoking Pack-Years	An equivalent numerical value of lifetime tobacco exposure. A pack year is defined as twenty cigarettes smoked every day for one year. (NA: Not Assessable) http://smokingpackyears.com/

**Table 3:** Terminology classification for tobacco users.

**Tobacco user category**	**Description**
Never-smoker	Refers to those who smoked <100 cigarettes in their lifetime and who, at the time of the diagnosis, did not smoke at all.
Former smoker	Refers to those who quit smoking for at least 1 year
Current smoker	Refers to those who were still smoking around the time of diagnosis.

**Table 4:** Tabular summary of IMRT characteristics.

**IMRT characteristics**	
Treatment duration in days; median (IQR)	43 (34–56)
Total prescribed dose in Grays; median (IQR)	70 (60–72)
Number of treatment fractions; median (IQR)	33 (30–35)
Dose per fraction in Grays; median (IQR)	2.12 (1.8–2.2)
Neck boost; n (%)	163 (60.6)

**Table 5:** Details of HPV status testing at diagnosis.

HPV DNA testing	
** Yes**	245 (85)
** No**	8 (2.8)
** N/A**	35 (12.2)
HPV DNA testing technique used	
** N/A**	4 (1.6)
** IHC**	2 (0.8)
** ISH**	239 (97.6)
P16 testing (IHC)	
** Yes**	222 (77)
** No**	31 (10.8)
** N/A**	35 (12.2)
**Patients tested for both HPV DNA & p16**	214 (74.3)

**Table 6:** DICOM PHI tags replaced with anonymized data.

**Tag (Group, Element)**	**Attribute name**
(0008,0012)	Instance creation date
(0008,0013)	Instance creation time
(0008,0020)	Study date
(0008,0021)	Series date
(0008,0022)	Acquisition date
(0008,0023)	Image date
(0008,0050)	Accession number
(0008,0081)	Institution address
(0008,0090)	Name of Physician
(0008,1010)	Station name
(0008,1040)	Institutional departmental name
(0010,0021)	Issuer of patient ID
(0010,0030)	Patient’s birth date
(0010,1040)	Patient’s address
(0020,0010)	Study ID
(0032,1032)	Requesting physician
(0040,0244)	Performed procedure step start date

**Table 7:** Description of data records uploaded to the figshare repository of the HPV and local recurrence prediction challenges ‘cited separately under (Data Citation 1) and (Data Citation 2).

**Data record**	**Description**
Training set of clinical meta-data.csv	A.csv file that encompasses clinical meta-data, regarding the patients assigned to the training set.
Training DICOM files set.zip	A compressed folder that contains anonymized DICOM files of the training data set. The subfolders are named according to each patient's ID after de-identification. Each individual patient's subfolder includes a DICOM file, named str.dcm, which comprises the set of segmented structures, i.e., primary and/or nodal disease.
Test set of clinical meta-data.csv	A.csv file that encompasses clinical meta-data, regarding the patients assigned to the test set.
Test DICOM files set.zip	A compressed folder that contains anonymized DICOM files of the test data set. The subfolders are named according to each patient's ID after de-identification. Each individual patient's subfolder includes a DICOM file, named str.dcm, which comprises the set of segmented structures, i.e., primary and/or nodal disease.
ReadMe.csv	Supplementary Information about the headings of the columns in the data sets.
Sample result submission.csv	A sample submission file in the correct format that includes an ID column like that in the solution file, plus a column with the predictions.
